# Oxidation behavior of AlN/CrN multilayered hard coatings

**DOI:** 10.1186/s40580-017-0109-y

**Published:** 2017-06-27

**Authors:** Darius Tytko, Pyuck-Pa Choi, Dierk Raabe

**Affiliations:** 1 Max-Planck-Institut für Eisenforschung GmbH, Max-Planck Str. 1, 40237 Düsseldorf, Germany; 2 Department of Materials Science and Engineering, Korea Advanced Institute of Science and Technology (KAIST), 291 Daehak-ro, Yuseong-gu, Daejeon, 305-338 Republic of Korea

**Keywords:** Oxidation, Hard coatings, Multi layers, Transmission electron microscopy, Atom probe tomography

## Abstract

We report on the oxidation behavior of AlN/CrN multilayers at 900 °C, deposited by radio frequency magnetron sputtering. It is shown that oxidation in this system is controlled by diffusion of Cr towards the surface and formation of Cr_2_O_3_. Cr diffusion is found to mainly occur along grain boundaries. Thus, coherent cubic AlN/CrN multilayer regions with coarse columnar grain structures are found to be oxidation resistant, whereas regions decomposed into hexagonal AlN/cubic CrN are prone to oxidation.

## Background

Oxidation is a commonly known process usually occurring at elevated temperatures, which often leads to severe surface degradation and to deterioration of material properties. It plays a particularly critical role in ceramic hard-coating materials, which are used for protection and property enhancement of cutting tools. In dry-cutting applications a protective coating is often exposed to temperatures as high as 1000 °C [1]. Hence, excellent oxidation resistance is a prerequisite for hard coating materials in order to preserve the mechanical properties, such as hardness and wear resistance, also at elevated temperatures. Recently it was reported that the CrAlN system exhibits both good mechanical properties and oxidation resistance [2–4]. The latter is due to the formation of a dense chemically stable oxide scale, which retards further oxidation by suppressing the supply of oxide-forming metallic elements [2, 5]. Coatings composed of alternating AlN/CrN layers show further enhancement of mechanical properties as compared to the CrAlN system [6–9]. Additionally, an improved oxidation resistance was observed in particular for AlN/CrN superlattice coatings with bilayer periods ≤4 nm [10–13]. An AlN layer thickness below 3 nm stabilizes the metastable cubic (c-AlN) crystal structure instead of the equilibrium hexagonal (h-AlN) structure [14–18]. The resulting superlattice coating has a specific microstructure with coherent c-AlN/CrN layers and strongly textured columnar grains [19]. Tien et al. [10] suggested that the columnar structure of the coating results in improved oxidation resistance. The purpose of this study is to elucidate the correlation between the microstructure of c-AlN/CrN coatings and the degree of oxidation and to reveal the diffusion processes occurring under exposure to air at 900 °C.

## Experimental

AlN/CrN multilayers were deposited by reactive radio frequency (RF) magnetron sputtering onto polished AISI 316 steel coupons. The sputtering chamber was evacuated to a pressure below 2 × 10^−7^ mbar, while the substrate was heated up to 350 °C. The used targets consisted of 99.9% pure Cr and 99.9% pure AlN and were 2 in. in diameter. The applied power at both targets was set to 100 W. Each target was equipped with a shutter system, which determines the layer thickness according to the opening time.

Initially, a 200 nm thick Cr layer was deposited as an adhesion layer. Subsequently, a 100 nm thick CrN buffer layer and alternating nanoscale AlN/CrN multilayers were deposited. For the deposition of the nitride phases a gas mixture of Ar and N_2_ with a ratio of 1:1 was used. The working pressure during all deposition processes prevailed at 3 × 10^−3^ mbar. The individual AlN and CrN layers were deposited by rotating the substrate back and forth between the AlN and Cr target, respectively.

A differential scanning calorimetry system was used for the annealing experiments. The coated steel coupons were heated up in the crucibles at a heating rate of 20 K/min, then held for 60 min at 900 °C and finally cooled down with 40 K/min. Flowing N_2_ gas mixed with ambient atmosphere was present during the annealing process.

Transmission electron microscopy (TEM) and energy dispersive X-ray spectroscopy (EDS) analyses were performed, using a JEOL JEM-2200FS operated at 200 kV.

TEM samples were prepared with a FEI Helios NanoLab 600i focus ion beam system using the standard lift-out technique [20, 21].

## Results and discussion

**Fig. 1:**
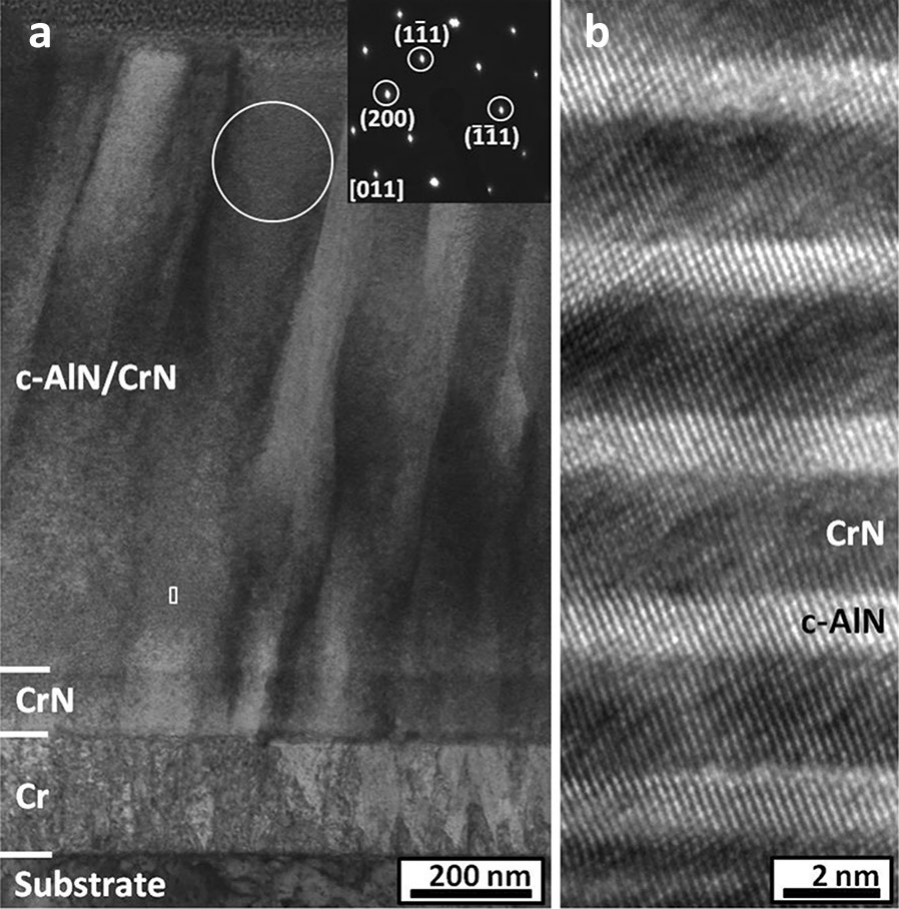
**a** STEM-BF cross section of the as-deposited coating showing columnar grains within the c-AlN/CrN region. The *inset* shows a SAED pattern taken from the grain marked by the *white circle*; **b** HR-TEM image of the c-AlN/CrN multilayers corresponding to the *white rectangle* in **a**. Coherency between the layers is evident

Figure 1a shows a cross sectional view of the as-deposited coating structure, as observed by scanning TEM (STEM). The microstructure consists of columnar grains and the total coating thickness is 1.2 µm. The inset in Fig. 1a shows a selected area electron diffraction pattern of one particular columnar grain marked by the circle. The pattern indicates that the B1 crystal structure continues throughout the entire AlN/CrN multilayer and that AlN prevails in a cubic crystal structure. High-resolution TEM investigations of the region marked with the white rectangle in Fig. 1a show the coherency of the multilayers (see Fig. 1b). The bright and dark contrast of the individual layers is attributed to the different scattering behavior of AlN and CrN. A layer thickness of 1.5 and 2.5 nm is measured for c-AlN and CrN, respectively.

**Fig. 2:**
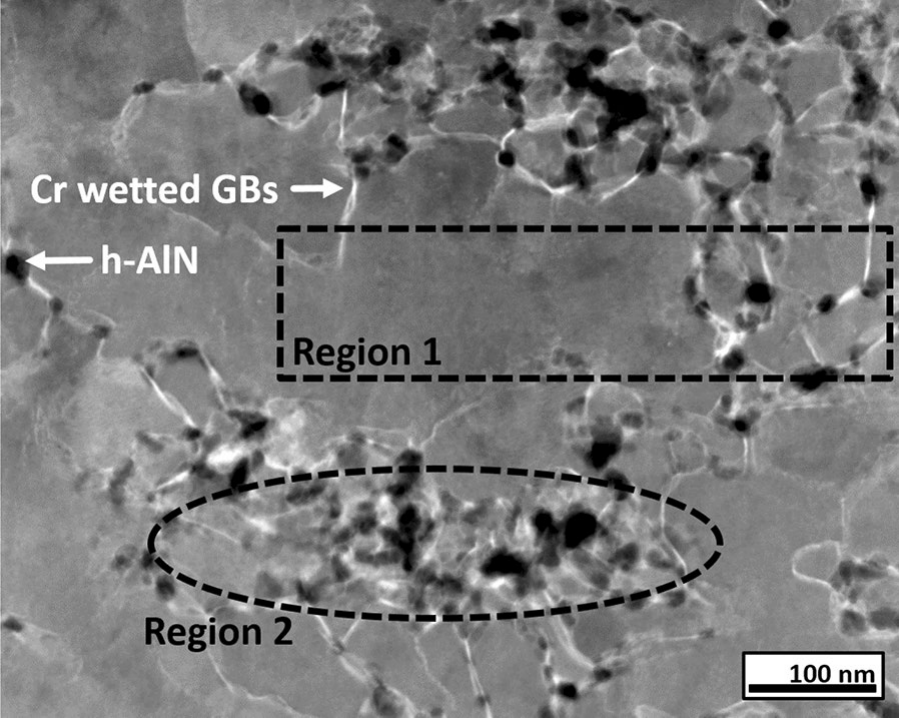
In-plain view of the c-AlN/CrN multilayers annealed for 15 min at 900 °C acquired by STEM-HAADF. The *bright lines* correspond to the CrN wetted columnar GBs. Hexagonal AlN (dark contrast) forms preferentially at GB junctions. Regions with low and high number densities of GB are apparent (Region 1 and 2, respectively)

Detailed analyses of the thermal stability and layer dissolution mechanisms of this coating are presented in [19]. One major finding of this coating after exposure to 900 °C is the interruption of the c-AlN layers at the grain boundaries (GBs). This effect is well known for immiscible multilayer systems and is often referred to as “pinch-off” [22–26]. The layer pinch-off is based on the ratio between the GB and interfacial energy, where the layer with the higher ratio is prone to thermal grooving at the GBs until it becomes completely interrupted. The higher ratio of the c-AlN as compared to CrN [19] yields the layer pinch-off at the GBs. As a consequence the columnar GBs become wetted by Cr. Furthermore, layer pinch-off at GB junctions leads to the formation of h-AlN predominantly at GB triple junctions, as verified in Ref. [19]. A high angle annular dark (HAADF) STEM image of an in-plain section of annealed c-AlN/CrN multilayers shows the microstructural changes described (see Fig. 2). The HAADF contrast is based on the atomic number Z, and thus, elements with higher Z appear brighter in this STEM mode. Consequently, the bright lines in Fig. 2 represent the CrN wetted GBs due to the higher atomic number Z of CrN as compared to AlN. In contrast, h-AlN precipitates formed at GB junctions are imaged as dark regions. Furthermore, Fig. 2 shows a strongly inhomogeneous grain size distribution in the range of 15–300 nm, resulting in regions with a low and high number density of GBs (compare Region 1 and 2 in Fig. 2, respectively).

**Fig. 3:**
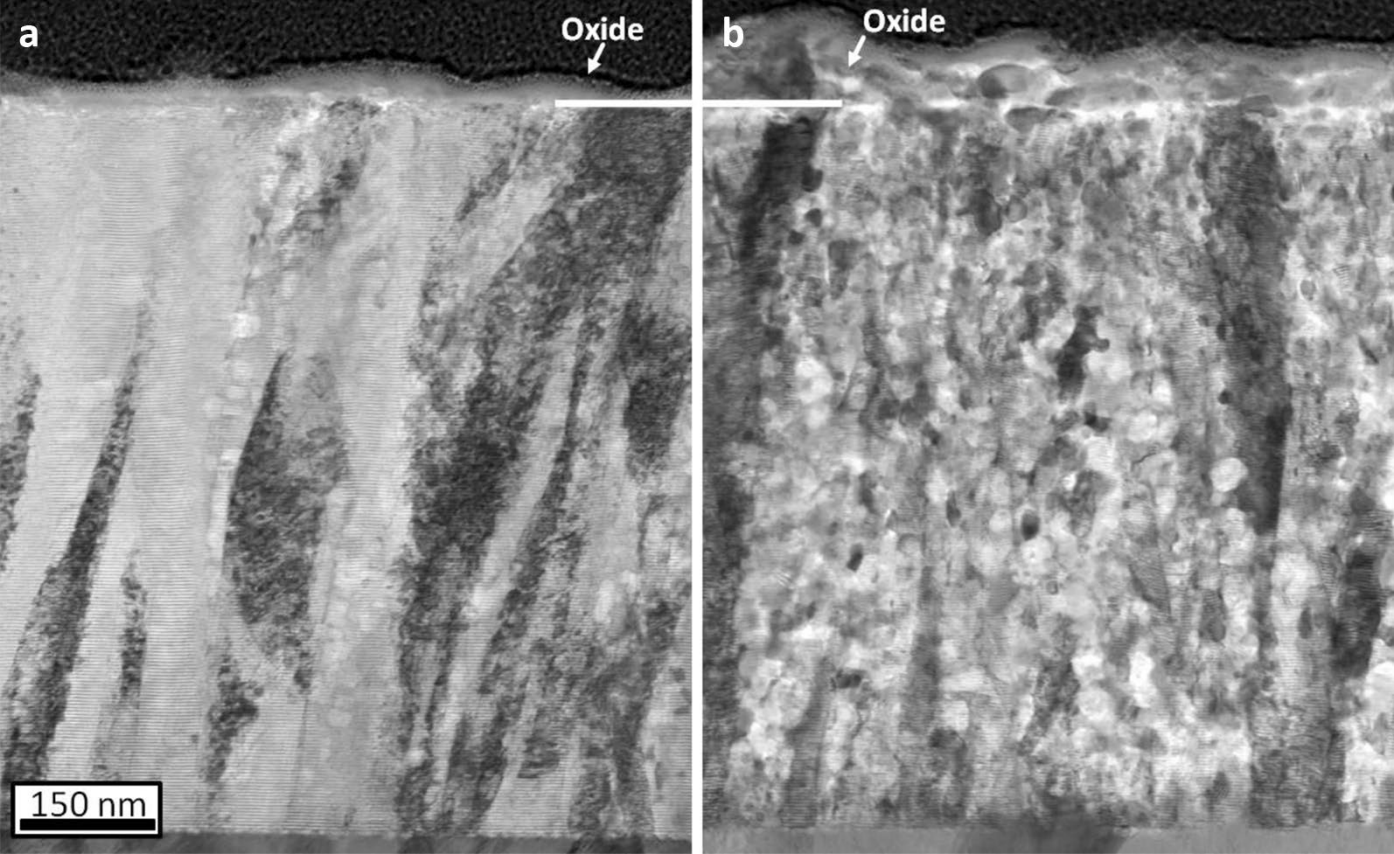
STEM-BF cross section views of the coating annealed for 60 min at 900 °C. TEM lamellas were prepared from different regions of the same sample containing a low (**a**) and high (**b**) number density of GB (compare Region 1 and 2 in Fig. 2). Regions with a high number of GBs are subject to pronounced oxidation

**Fig. 4:**
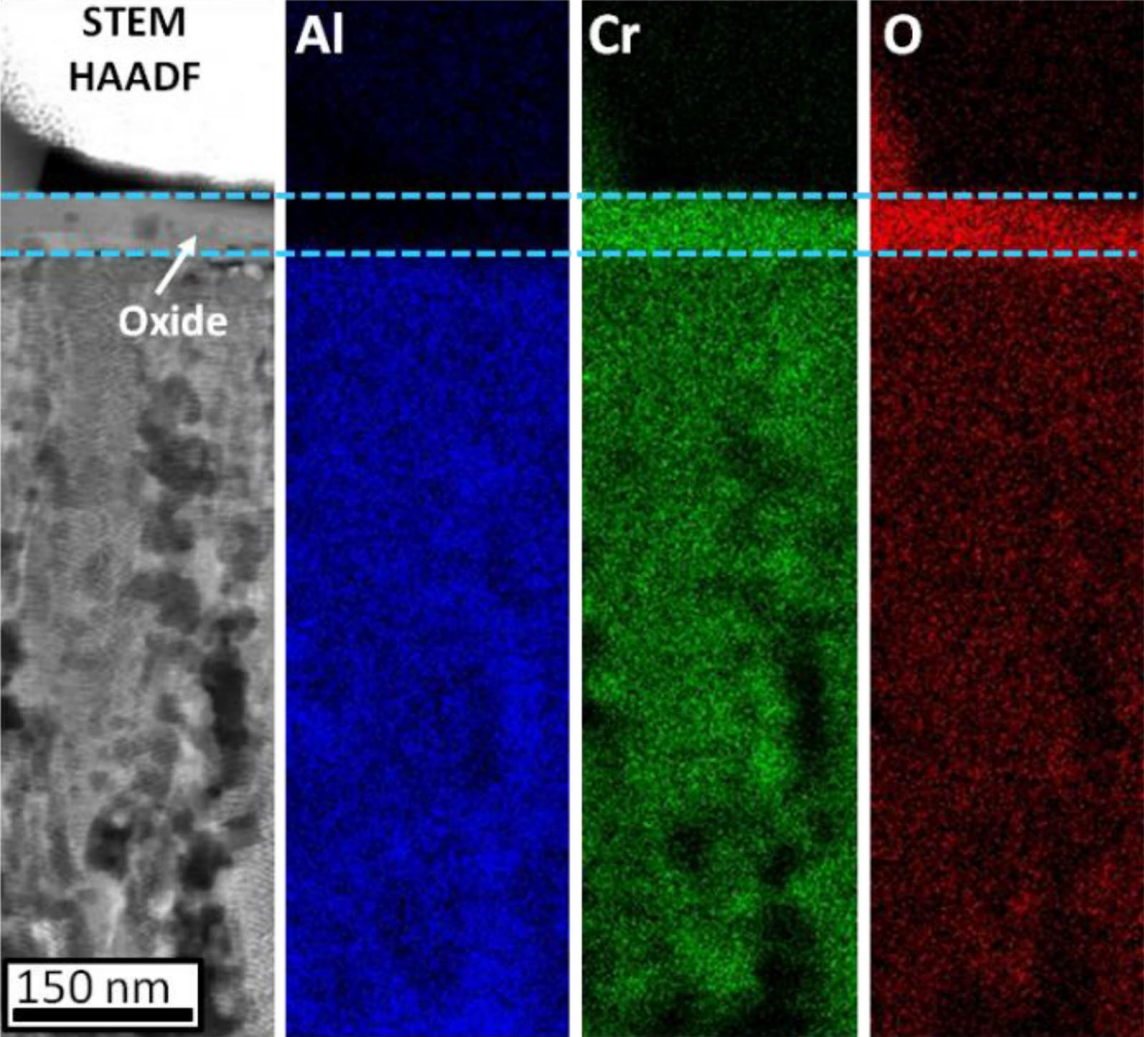
STEM HAADF and EDS mappings of the c-AlN/CrN superlattice annealed for 60 min at 900 °C

In order to study the local oxidation behavior of the coating, cross sectional STEM analyses were performed at regions with low and high GB densities of the same sample after annealing at 900 °C for 60 min (see Fig. 3). A region with a low number density of GBs and thus large columnar grains is presented in Fig. 3a. This region shows predominantly undisturbed multilayers and a small number of h-AlN precipitates at the GB junctions. In contrast, a region with a high number density of GBs is presented in Fig. 3b. Highly disturbed multi layers and a large number of h-AlN are present. Both regions show a strongly different oxidation behavior in terms of the resulting oxide layer thickness (see oxide in Fig. 3a, b). As can be seen from cross-sectional TEM images and EDX maps (see Fig. 4), oxidation appears to occur via external oxidation and not by internal oxidation, i.e. by transport of metal atoms to the surface rather than by transport of oxygen atoms into the bulk. Also, oxide formation is significantly influenced by the coating microstructure, since the low and high GB density regions form oxides with ~10 and ~100 nm in thickness, respectively. As outward diffusion of the oxide-forming element Cr is enhanced along the grain boundaries, the surface region above the high GB density region shows a large oxide layer thickness.

Previous studies by Ichimura et al. [27, 28] on the oxidation of AlN and CrN coatings indicated that oxidation in the AlN/CrN system is controlled by the outward diffusion of Cr due to its lower activation energy for diffusion in Cr_2_O_3_ (255 kJ/mol [27]) as compared to diffusion of Al in Al_2_O_3_ (477 kJ/mol [28]). The experimental results obtained in this work are in good agreement with those findings. STEM-EDS maps of the sample annealed at 900 °C for 60 min show a continuous Cr-rich oxide layer (see Fig. 4). The exact composition of the oxide was determined by APT and yields O–35.5Cr–6.9Al–4.8N (in at.%).

Using atom probe tomography, we previously showed that the AlN layers within the AlN/CrN multi layers are chemically stable [19]. For instance, the AlN layers maintain their chemical stoichiometry in all annealing conditions and show a low solubility for Cr (~4.5 at.% after 60 min at 900 °C). Hence, these layers act as an effective barrier for Cr outward diffusion resulting in significantly thinner Cr_2_O_3_ oxides as compared to CrN single layer coatings [29]. Investigations on the oxidation resistance of AlN/CrN multilayers with different modulation periods showed improved oxidation resistance with decreasing bilayer period [8, 10, 29, 30]. Since the AlN layers act as diffusion barriers the transport of Cr towards the surface occurs mainly along crystallographic defects such as GBs and incoherent h-AlN/CrN interfaces. This assumption is supported by the fact that CrN films form after heat treatment at AlN GBs as detected by STEM-HAADF and APT.

Hence, c-AlN/c-CrN multilayers with sufficiently thin AlN layers show an improved oxidation resistance due to the stabilization of AlN to the cubic crystal structure, which becomes coherent with the CrN layers. In this specific microstructure only the columnar GB contribute to the outward diffusion of Cr. The limited diffusion paths of Cr retard the supply of the oxide-forming element on the surface.

In contrast, the microstructure of the h-AlN/CrN system consists of equiaxed grains within each layer and does not exhibit a columnar grain structure. In this case both, the GBs as well as the incoherent interfaces act as possible diffusion paths for Cr diffusion. It should also be noted that the formation of the h-AlN precipitates at the GB junctions induces severe plastic distortions due to a volume expansion of about 30% yielding a significant number of defects to the surrounding multilayers. These defects enhance the diffusion of elements and may even form additional GBs which increase the outward diffusion of Cr.

The degree of oxidation in c-AlN/CrN superlattice coatings depends on the number density of columnar GBs and hence the number of escape routes for Cr. As GB triple junctions promote the formation of the h-AlN phase [19], regions with a high number of GBs enable a faster transport of Cr towards the surface. Consequently, such regions exhibit thicker oxide layers as compared to regions with lower GB density. The restricted number of Cr diffusion paths within c-AlN/CrN superlattices yields a deficiency of the oxide-forming element at the surface. In other words, the oxidation rate is slowed down and results in a superior oxidation resistance.

## Conclusion

The results obtained in this study show that the microstructure strongly determines the oxidation behavior of AlN/CrN multilayer coatings at elevated temperatures (900 °C/60 min). The transport of Cr towards the surface controls the formation of Cr_2_O_3_, whereas the diffusion occurs mainly along GBs and incoherent interfaces such as h-AlN/CrN. Therefore, c-AlN/CrN multilayer coatings with coherent interfaces show an improved oxidation resistance due to a lower number of Cr diffusion paths. Within the coherent system different degrees of oxidation were found which are attributed to the number density of GBs and the local microstructure of the coating. In particular, reduced oxidation is observed for regions with large columnar grains.

Consequently, c-AlN/CrN coatings with coherent multilayers and a low number of GBs have a superior oxidation resistance. An optimized deposition process should be applied in order to achieve coatings with such a desired microstructure. Alternatively, GB engineering could be applied to lower the GB energy of the c-AlN layers by decoration with solute elements. This could suppress the GB pinch-off and thus avoid GB wetting by Cr. As a result, the Cr transport towards the surface would be decelerated and the oxide growth retarded.
